# Individual and area-level socioeconomic correlates of hypertension prevalence, awareness, treatment, and control in uMgungundlovu, KwaZulu-Natal, South Africa

**DOI:** 10.1186/s12889-023-15247-0

**Published:** 2023-03-02

**Authors:** SLM Madela, NW Harriman, R Sewpaul, AD Mbewu, DR Williams, S Sifunda, T Manyaapelo, A Nyembezi, SP Reddy

**Affiliations:** 1 Expectra Health Solutions, Dundee, South Africa; 2 Social and Behavioral Sciences Department, Harvard T.H. Chan School of Public Health, Boston, USA; 3 Human and Social Capabilities Division, Human Sciences Research Council, Pretoria, South Africa; 4 Sefako Makgatho Health Sciences University, Ga-Rankuwa, South Africa; 5 African and African American Studies Department, Harvard University, Cambridge, USA; 6 Africa Health Research Institute, Somkhele, South Africa; 7 University of the Western Cape, Cape Town, South Africa; 8 University of KwaZulu-Natal, Berea, South Africa

**Keywords:** Hypertension, Black South Africans, Socioeconomic status, Area-level deprivation

## Abstract

**Background:**

Hypertension is the second leading risk factor for death in South Africa, and rates have steadily increased since the end of Apartheid. Research on the determinants of hypertension in South Africa has received considerable attention due to South Africa’s rapid urbanization and epidemiological transition. However, scant work has been conducted to investigate how various segments of the Black South African population experience this transition. Identifying the correlates of hypertension in this population is critical to the development of policies and targeted interventions to strengthen equitable public health efforts.

**Methods:**

This analysis explores the relationship between individual and area-level socioeconomic status and hypertension prevalence, awareness, treatment, and control within a sample of 7,303 Black South Africans in three municipalities of the uMgungundlovu district in KwaZulu-Natal province: the Msunduzi, uMshwathi, and Mkhambathini. Cross-sectional data were collected on participants from February 2017 to February 2018. Individual-level socioeconomic status was measured by employment status and educational attainment. Ward-level area deprivation was operationalized by the most recent (2011 and 2001) South African Multidimensional Poverty Index scores. Covariates included age, sex, BMI, and diabetes diagnosis.

**Results:**

The prevalence of hypertension in the sample was 44.4% (*n* = 3,240). Of those, 2,324 were aware of their diagnosis, 1,928 were receiving treatment, and 1,051 had their hypertension controlled. Educational attainment was negatively associated with hypertension prevalence and positively associated with its control. Employment status was negatively associated with hypertension control. Black South Africans living in more deprived wards had higher odds of being hypertensive and lower odds of having their hypertension controlled. Those residing in wards that became more deprived from 2001 to 2011 had higher odds of being aware of their hypertension, yet lower odds of receiving treatment for it.

**Conclusions:**

Results from this study can assist policymakers and practitioners in identifying groups within the Black South African population that should be prioritized for public health interventions. Black South Africans who have and continue to face barriers to care, including those with low educational attainment or living in deprived wards had worse hypertension outcomes. Potential interventions include community-based programs that deliver medication to households, workplaces, or community centers.

**Supplementary Information:**

The online version contains supplementary material available at 10.1186/s12889-023-15247-0.

## Background

Noncommunicable diseases (NCDs) are the leading cause of mortality worldwide. Collectively, NCDs, including cardiovascular disease, cancers, respiratory disease, and diabetes account for more than 71% of global deaths [1]. The burden of NCD mortality is disproportionately borne by low- and middle-income countries (LMIC), with 77% of deaths occurring in these nations. Deaths due to cardiovascular disease (CVD), which include heart and cerebrovascular diseases, make up the largest proportion of NCD deaths (43%) and are similarly patterned by economic development – over 75% of these deaths also occur in LMICs [1].

One such nation is South Africa, which has received increased attention in scientific research due to its rapid urbanization and progression through the epidemiological transition over the past 30 years [2, 3]. In South Africa, recent estimates suggest that NCDs account for 43% of deaths, 18% of which are due to CVD [4]. Hypertension is a well-documented and preventable risk factor for CVD and other NCDs, such as cerebrovascular accident, myocardial infarction, congestive heart failure, and kidney injury. It is the second leading risk factor for death in South Africa, and nationally-representative estimates have indicated a marked increase in hypertension prevalence in the past two decades [5–7]. The national prevalence of hypertension has more than doubled from the first Demographic and Health Survey (DHS) in 1998 (23%) to the most recent data from 2016 (48.3%) [8–10].

Hypertension management strategies have typically been informed by estimates of the hypertension care cascade, which includes hypertension screening, prevalence, awareness, treatment, control, and their correlates. For this management to be effective, both the healthcare workers and the patients need to adhere to the treatment guidelines and prescribed treatment respectively [11]. As the prevalence of hypertension in South Africa has increased, large gaps in this care cascade have emerged. National data from the 2011–2012 South African National Health and Nutrition Examination Survey (SANHANES) indicated that half of all hypertensive individuals were unaware of their condition and only 8.9% had their hypertension controlled [12]. More recent estimates from probability samples have revealed care continuum gaps of similar magnitude. Data from the second wave of the World Health Organization’s Study on Global Ageing (WHO-SAGE) obtained in 2015 showed that 58% of hypertensive adults were unaware of their condition and 18% had their hypertension controlled [13]. The most recent nationally representative estimates, obtained from the 2016 DHS, confirmed these gaps, with 45% of hypertensive individuals unaware of their condition, and 16% controlled [9]. The early detection and control of hypertension are vitally important, firstly to reduce the rates of CVD and secondly to decrease the incidence and burden of other NCDs described above.

South Africa’s epidemiological transition and urbanization have led the burden of NCDs to impact groups differently, based on individual and area-level measures of socioeconomic status (SES) [14–18]. Interestingly, recent nationally representative analyses have revealed complex patterns in the association between SES and hypertension. According to an analysis from the National Income Dynamics Survey (NIDS), educational attainment was only associated with hypertension control in females, while income was associated with control in males [15]. Analyses from the 2016 DHS have revealed a similar divergence between income and education with educational attainment being inversely related to hypertension but household wealth positively associated with elevated risk in bivariate analyses [9]. Data from the 2015 WHO-SAGE had similar findings for education but did not include a measure of household income or wealth [13]. Furthermore, the reported positive association between urban residence and hypertension in this study is inconsistent with other nationally representative analyses – including the 2011–2012 SANHANES, which found that hypertension prevalence was higher in rural residential settings [12]. It is well-documented that the epidemiological transition occurs at varying rates across segments of a population [19, 20]. In South Africa, the unclear direction of the socioeconomic gradient with hypertension may be due to a limitation of aggregate data to account for different sub-populations experiencing the transition at varying rates. As such, it is important to investigate these sub-populations to better understand where they lie within the transition; and to tailor public health prevention efforts accordingly.

South Africa’s historical context informs which sub-populations should be prioritized for epidemiologic inquiry. The effects of discriminatory Apartheid-era policies manifest themselves in marked racial inequality in the present day. One such policy, the Group Areas Act, created mass racial segregation across most areas in South Africa; restricting employment and educational opportunities for generations – resulting in vast racial disparities in individual-level SES (i.e., income, household wealth, and educational attainment) today [21–23]. Furthermore, such policies presented geographical barriers that limited access to desirable societal resources, including healthcare institutions (i.e., primary health centers, and outpatient health centers), which contributed to area-level deprivation, the effects of which are disproportionately experienced by Black South Africans [24, 25]. In tandem, these inequities impact Black South Africans’ proximity to care, awareness of health-promoting behaviors, and ability to afford care.

While nationally representative data does not currently suggest that Black South Africans experience disproportionately high levels of hypertension at the national level in South Africa, existing research has suggested that this may be due to the Black South African population being in an earlier stage in the epidemiological transition, compared to other racial groups [26]. The aforementioned individual and area-level socioeconomic inequities resulting from Apartheid-era policies have the potential to adversely affect the hypertension care continuum within this population as it undergoes the epidemiological transition. As the Black South African population progresses through the transition, information on the correlates of the prevalence of hypertension and its care cascade can be of immense value, to better inform initiatives and policies to alleviate the burden of disease.

Compared to the plethora of empirical research on the determinants of hypertension at an aggregate population level, scant attention has been given to the determinants within the Black South African sub-population. Indeed, Black South Africans are often treated as a homogenous group in the scientific literature, despite the documented geographic, cultural, and socioeconomic variation within the population [7]. Few studies in South Africa have attempted to highlight these differences and investigate their relation to hypertension. The sparse extant work that does focus on this subpopulation’s different geographic and socioeconomic patterns and their relation to hypertension was conducted over a decade ago and requires revisiting [27–30]. Furthermore, recent work has concentrated on the associations between hypertension and clinical factors, such as the presence of comorbidities, and modifiable behaviors, such as diet and alcohol intake, rather than on socioeconomic determinants [31–33].

Understanding the socioeconomic heterogeneity within the Black South African sub-population and its associations with hypertension is critical to identifying the risk factors for hypertension that exist in a population that is progressing through the epidemiological transition. This information is vital to the development of policies and targeted interventions to bolster public health efforts. To that end, this paper aims to explore the relationship between individual and area-level SES, including change in area-level deprivation over time, and hypertension prevalence, awareness, treatment, and control within a sample of Black South Africans in the Umgungundlovu district of KwaZulu Natal, South Africa.

## Methods

### Data source

Data were analyzed from the HealthRise South Africa Study, a community-based project designed to improve screening, diagnosis, management, and control of hypertension and diabetes in underserved communities [34]. The HealthRise Study was sponsored by the Medtronic Foundation and implemented by Expectra Health Solutions; a non-governmental organization (NGO) accredited by the Health & Welfare Sector Education Training Authority (SETA) in Level 4 community health work. The National Qualification Framework is used to measure the skills and knowledge of learners in South Africa; it ranges from 1 to 10, with higher values indicating greater mastery of knowledge. A Level 4 certificate is equivalent to what students in South Africa receive after grade 12, and Expectra Health Solutions is accredited to confer this level of expertise to its community health workers (CHWs). Cross-sectional data were collected from February 2017 to February 2018 from three local municipalities of the uMgungundlovu district in KwaZulu-Natal province: the Msunduzi, uMshwathi, and Mkhambathini. The district and its local municipalities were purposively selected in a consultative process with the KwaZulu-Natal Department of Health and the Institute for Health Metrics and Evaluation (IHME). Eligible catchment areas included those with minimal or no existing Department of Health CHWs. The selected areas were also required to include residents living in rural informal, rural formal (farm), urban formal, and urban informal areas. The final selected areas had a catchment population of 163,995. More detail on the eligibility criteria and consultative process is described in detail elsewhere [35–38].

Data collection was implemented by twenty-five CHWs trained by Expectra Health Solutions to adhere to the KwaZulu-Natal Department of Health Adult Primary Care (APC) guidelines for diagnosing hypertension and diabetes [39, 40]. Data were collected over the study period through three methods: door-to-door household visits, community outreach campaigns, and workplace visits. Overall, 7,954 people consented to be screened from the 10,658 door-to-door household visits. Five hundred and twenty-five people consented to be screened from the sixteen workplace visits. Finally, 2,353 people were screened from 24 community outreach campaigns that took place over the study period. The analyses presented in this paper were restricted to Black South Africans who were 18 years and older and whose data were obtained during door-to-door survey visits (*n* = 7,303). This restricted sample was chosen because information on participant SES and area-level data were only collected in the surveys used during the door-to-door data collection process.

### Dependent variables

This analysis included four dichotomous hypertension-related dependent variables: hypertension prevalence, awareness, treatment, and control. Blood pressure was measured using digital blood pressure machines following the KwaZulu-Natal Department of Health APC guidelines. Specifically, patients were seated and had their arms supported at heart-level for 3–5 min. Two measurements were obtained 1–2 min apart, and if these readings differed by more than 5 mmHg, a third reading was obtained. If any of the measurements were over 140/90 mmHg, the patient had their blood pressure confirmed 6 – 24 h later.

Individuals were defined as hypertensive if their measured blood pressure at the time of the study was confirmed to be ≥ 140/90 mmHg, if they indicated they had been previously diagnosed with hypertension by a healthcare professional, or if they reported taking anti-hypertensive medication. Participants were defined as aware of their hypertension if they were 1) hypertensive, and 2) reported that a healthcare professional had previously diagnosed them. Hypertensive treatment was conditional upon hypertension awareness – subjects were considered in treatment if they were 1) aware of their hypertension and 2) reported that they were currently receiving anti-hypertensive medication for it. Finally, subjects were considered in control of their hypertension if they 1) were currently receiving treatment for their hypertension, and 2) their measured blood pressure at the time of the study was below 140/90 mmHg.

### Individual-level independent variables

Demographic variables included age (18–30, 31–60, over 60) and sex (male vs. female). Regarding the proxies for SES, the education variable had five categories (none, some primary school, incomplete high school, completed high school, and tertiary). Employment status was self-reported. When possible, unemployment was verified by confirming that the individual received unemployment benefits from the South African government. Individuals who indicated they were self-employed were categorized as employed, and our final variable was dichotomous (unemployed vs. employed). The body mass index (BMI) variable had four categories: underweight (< 18.5 kg/m^2^), normal weight (18.5–24.9 kg/m^2^), overweight (25–29.9 kg/m^2^), and obese (≥ 30 kg/m^2^). The KwaZulu-Natal Department of Health APC guidelines were followed to diagnose individuals with diabetes. Blood was obtained from a finger prick, and individuals were defined as having diabetes if their level in a random blood glucose test at the time of the examination was 11.0 mmol/L or higher or if they reported a healthcare professional had previously diagnosed them with diabetes.

### Area-level independent variables

Geographic data at the municipality and ward levels were also collected as part of the HealthRise project. To assess area-level deprivation, we linked this information to the 2011 (most recently available) and 2001 census multidimensional poverty data. We utilized the South African Multidimensional Poverty Index (SAMPI), which was developed by Statistics South Africa to measure area-level deprivation unique to the South African context [41]. SAMPI scores range from 0 to 1 with higher scores indicating greater area-level deprivation. The SAMPI is comprised of four household indicators of deprivation – education, health, the standard of living, and economic activity – the specific cut points and weights of these are described elsewhere [41]. The SAMPI score for a geographic area is the product of two metrics: 1) the poverty headcount – the proportion of households in the area that are deprived on at least one of the four indicators, and 2) the poverty intensity – the average proportion of the four indicators that deprived households are deprived in. For example, if 50% of the households in an area were deprived, and the deprived households were deprived on three of the four indicators (75%), on average, the SAMPI score for this area would be 0.5*0.75 = 0.375.

The SAMPI has been linked in prior research to various dimensions of health, including self-rated health and cardiovascular disease [42, 43]. It has typically been modeled as a categorical variable with cut points based on the quintiles of the distribution of scores. In this study, SAMPI scores were modeled as a categorical variable with cut points based on the quintiles of the distribution of SAMPI scores for all of South Africa, giving us an indication of how deprived the areas from which we obtained our sample were. Using this methodology, we computed SAMPI quintile variables for 2001 and 2011. We also constructed a variable to determine if a ward’s relative SAMPI quintile had changed from 2001 to 2011.

### Statistical analyses

First, we calculated descriptive statistics for the dependent and independent variables (unweighted percentages) including missing values. To maintain the power of the analyses, we imputed missing values for all the variables included in the regression models using multiple imputation with chained equations. We produced and performed analyses on 10 imputations of the dataset. We also conducted sensitivity analyses of our regression models including only those with no missing data to ensure the robustness of our reported results to missing data, and our results were consistent with the imputed models.

Following the imputation procedure, we conducted a series of six hierarchical logistic regression models for each of the four hypertension outcomes (Prevalence, Awareness, Treatment, and Control) using Stata 15.0. Model 1 estimated the association between the hypertension outcome of interest and demographic variables (age and sex). Model 2 added variables that captured individual-level markers of SES (education level and employment status). Model 3 included area-level deprivation measures to model 2. Finally, in model 4, individual risk factors (BMI and diabetes prevalence) were added to model 3. For each hypertension outcome, models 3 and 4 were each run with an “a” and “b” counterpart for our different measures of area-level deprivation – “a” models used the 2011 SAMPI quintile, while “b” models used the change in SAMPI quintile from 2001 to 2011.

## Results

### Sample description

In Table 1, we present the descriptive statistics of the study sample. The majority of the sample was between the ages of 31 and 60 (53.9%). There was a high proportion of females in the sample (72.4%). Regarding the markers of SES, many of the respondents had no formal education (32.5%); the next highest percentage was those who had completed high school (23.3%). Five percent of the sample had attained a tertiary education level. The majority of the sample was unemployed (70.7%). Eleven percent of the sample was underweight (BMI: < 18.5 kg/m^2^), 38% of the sample had normal weight (18.5–24.9 kg/m^2^); 22% were overweight (25–29.9 kg/m^2^); and 24% were obese (> = 30 kg/m^2^). The prevalence of diabetes in the sample was 13.9%.

**Table 1:** Sample Description

	**N**	**Percentage**
***Hypertension***		
**Hypertension Prevalence**		
No current or previous hypertension	4,063	55.6%
Current or previous hypertension	3,240	44.4%
Missing	0	0.0%
**Hypertension Awareness**		
Unaware	916	28.3%
Aware	2,324	71.7%
Missing	0	0.0%
**Hypertension Treatment**		
Untreated	396	17.0%
Treatment	1,928	83.0%
Missing	0	0.0%
**Hypertension Control**		
Uncontrolled	877	45.5%
Controlled	1,051	54.5%
Missing	0	0.0%
***Demographics***		
**Age**		
18–30	1,709	23.4%
31–60	3,938	53.9%
Over 60	1,656	22.7%
Missing	0	0.0%
**Sex**		
Male	2,016	27.6%
Female	5,287	72.4%
Missing	0	0.0%
***Socioeconomic Status***		
**Education Level**		
None	2,375	32.5%
Some Primary School	1,384	19.0%
High School Incomplete	1,244	17.0%
High School Complete	1,701	23.3%
Tertiary	366	5.0%
Missing	233	3.2%
**Employment Status**		
Unemployed	5,162	70.7%
Employed	1,858	25.4%
Missing	283	3.9%
***Risk Factors***		
**Body Mass Index**		
Underweight < 18.5 kg/m2	813	11.1%
Normal weight 18.5–24.9 kg/m2	2,744	37.6%
Overweight 25–29.9 kg/m2	1,622	22.2%
Obese > = 30 kg/m2	1,767	24.2%
Missing	357	4.9%
**Diabetes**		
No previous or current diabetes diagnosis or not eligible for screening	6,291	86.1%
Current or previous diabetes	1,012	13.9%
Missing	0	0.0%
***Area Deprivation***		
**2011 Deprivation Quintile**		
Q1 Least Deprived	0	0%
Q2	182	2.5%
Q3	1,664	22.8%
Q4	4,559	62.4%
Q5 Most Deprived	0	0%
Missing	898	12.3%
**Deprivation Quintile Change (2001 to 2011)**		
No change	5,525	75.7%
Quintile Worsened	880	12.1%
Missing	898	12.3%

Regarding the area-level measures of deprivation, most of the sample lived in Wards in the 4^th^ Quintile of South Africa’s 2011 deprivation score distribution (62.4%). No respondents lived in Wards that were in the 1^st^ (least deprived) or 5^th^ (most deprived) quintiles of South Africa’s 2011 area deprivation distribution. The majority of respondents lived in Wards with deprivation levels that did not change from 2001 to 2011 (75.7%). None of the respondents lived in Wards whose deprivation quintile improved from 2001 to 2011 (i.e., areas that became less deprived). Detailed descriptions of the geographic variables and linked census data can be found in supplementary material 1.

The prevalence (current or previous diagnosis) of hypertension in the sample was 44.4% (*n* = 3,240). Of those who were hypertensive, 71.7% were aware of their diagnosis (*n* = 2,324). Among those aware, 83% were currently receiving treatment for their hypertension (*n* = 1,928). Finally, of those who were receiving treatment, 54.5% had their hypertension controlled (*n* = 1,051).

### Logistic regression models

#### Hypertension prevalence

Table 2 presents the results from the hierarchical logistic regression models for hypertension prevalence among Black South African adults (*n* = 7,303). Across all models, we observed a positive association between age and hypertension prevalence, and that sex was not associated with hypertension prevalence.

**Table 2:** Odds Ratios (OR) and Standard Errors (SE) for Hierarchical Logistic Regression Models for Hypertension Prevalence (*n* = 7,303)

**Hypertension Prevalence**	Model 1: Age + Sex		Model 2: Model 1 + SES		Model 3a: Model 2 + Area Deprivation Quintile		Model 4a: Model 3a + BMI and DM Prevalence		Model 3b: Model 2 + Area Deprivation Change		Model 4b: Model 3b + BMI and DM Prevalence	
**Variables**	OR	SE	OR	SE	OR	SE	OR	SE	OR	SE	OR	SE
**Age**												
31–60	*Ref*		*Ref*		*Ref*		*Ref*		*Ref*		*Ref*	
18–30	0.324 **	0.023	0.366 **	0.027	0.372 **	0.027	0.429 **	0.032	0.366 **	0.027	0.421 **	0.032
Over 60	4.869 **	0.328	4.021 **	0.288	3.997 **	0.287	3.563 **	0.269	4.023 **	0.289	3.585 **	0.270
**Sex**												
Male	*Ref*		*Ref*		*Ref*		*Ref*		*Ref*		*Ref*	
Female	1.111	0.064	1.083	0.064	1.092	0.064	0.946	0.060	1.082	0.064	0.942	0.059
**Education**												
None			*Ref*		*Ref*		*Ref*		*Ref*		*Ref*	
Some primary education			1.150	0.085	1.156	0.086	1.160	0.090	1.150	0.085	1.145	0.089
High school incomplete			0.778 **	0.060	0.777 **	0.060	0.766 **	0.062	0.779 **	0.060	0.763 **	0.062
High school complete			0.611 **	0.046	0.603 **	0.046	0.631 **	0.050	0.611 **	0.046	0.635 **	0.050
Tertiary			0.604 **	0.080	0.589 **	0.078	0.574 **	0.079	0.606 **	0.080	0.592 **	0.081
**Employment Status**												
Unemployed			*Ref*		*Ref*		*Ref*		*Ref*		*Ref*	
Employed			0.885	0.056	0.882 *	0.056	0.912	0.060	0.882 *	0.056	0.919	0.061
**2011 Area Deprivation Quintile**												
Q2					*Ref*		*Ref*					
Q3					1.818 **	0.353	1.863 **	0.387				
Q4					2.014 **	0.375	2.252 **	0.444				
**Body Mass Index**												
Normal weight 18.5–24.9 kg/m2							*Ref*				*Ref*	
Underweight < 18.5 kg/m2							0.898	0.086			0.896	0.086
Overweight 25–29.9 kg/m2							1.141	0.083			1.131	0.082
Obese > = 30 kg/m2							1.479 **	0.107			1.456 **	0.105
**Diabetes**												
No current or previous diabetes diagnosis							*Ref*				*Ref*	
Current or previous diabetes diagnosis							7.840 **	0.799			7.747 **	0.788
**2001 to 2011 Area Deprivation Quintile Change**												
No change in quintile									*Ref*		*Ref*	
Quintile worsened									1.038	0.082	0.942	0.078

^*^ *p* < 0.05

^**^ *p* < 0.01

Model 2 considers the role of individual-level indicators of SES (educational attainment and employment status). Compared to Black South Africans with no formal education, those with some high school education (OR 0.778, *p* = 0.001), as well as those who completed high school education (OR 0.611, *p* < 0.001), and those with tertiary education (OR 0.604, *p* < 0.001) had lower odds of hypertension.

In Model 3a, adults living in more deprived areas had higher odds of being hypertensive (3^rd^ quintile: OR 1.818, *p* = 0.002 and 4^th^ quintile: OR 2.014, *p* < 0.001), compared to those in the least deprived wards (2^nd^ quintile). The consideration of area derivation did not materially alter the graded negative association between educational attainment and hypertension but a significant difference in the odds of being hypertensive for employed adults compared to the unemployed (OR 0.882, *p* = 0.048) emerged once area deprivation was added to the model.

In model 4a, as expected, both BMI and a history of diabetes are significantly associated with increased hypertension risk. However, the consideration of these risk factors reduced the association of employment status to non-significance but did not lead to a change in the association of education. The hypertension disparities linked to area deprivation widened in this model (Q3 vs Q2: OR 1.863, *p* = 0.003; Q4 vs Q2: OR 2.252, *p* < 0.001).

In Model 3b, change in area deprivation over 10 years is unrelated to hypertension prevalence. However, as noted in model 3a, after adjustment for area deprivation changes, the association between educational attainment and hypertension prevalence remained evident and, additionally, employment status emerged as significant with employed adults having lower odds of hypertension than those who were unemployed (OR 0.882, *p* = 0.049).

In model 4b, change in area deprivation is not significantly associated with hypertension prevalence, and with the inclusion of BMI and diabetes in this model, while employment status is no longer associated with hypertension prevalence, education remains inversely related to hypertension.

#### Hypertension awareness

In Table 3, we present the results of the logistic regression models for hypertension awareness, in which the sample is restricted to those who are currently hypertensive (*n* = 3,240). In model 1, and all subsequent models, we observed a positive association between age and hypertension awareness, and females had significantly higher odds of hypertension awareness than men.

**Table 3:** Odds Ratios (OR) and Standard Errors (SE) for Hierarchical Logistic Regression Models for Hypertension Awareness (*n* = 3,240)

**Hypertension Awareness**	Model 1: Age + Sex		Model 2: Model 1 + SES		Model 3a: Model 2 + Area Deprivation Quintile		Model 4a: Model 3a + BMI and DM Prevalence		Model 3b: Model 2 + Area Deprivation Change		Model 4b: Model 3b + BMI and DM Prevalence	
**Variables**	OR	SE	OR	SE	OR	SE	OR	SE	OR	SE	OR	SE
**Age**												
31–60	*Ref*		*Ref*		*Ref*		*Ref*		*Ref*		*Ref*	
18–30	0.443 **	0.057	0.509 **	0.068	0.502 **	0.067	0.561 **	0.080	0.501 **	0.067	0.560 **	0.080
Over 60	2.967 **	0.287	2.525 **	0.261	2.533 **	0.263	2.460 **	0.267	2.564 **	0.265	2.485 **	0.270
**Sex**												
Male	*Ref*		*Ref*		*Ref*		*Ref*		*Ref*		*Ref*	
Female	2.667 **	0.238	2.626 **	0.237	2.557 **	0.232	2.220 **	0.219	2.585 **	0.234	2.233 **	0.220
**Education**												
None			*Ref*		*Ref*		*Ref*		*Ref*		*Ref*	
Some primary education			0.885	0.104	0.872	0.102	0.876	0.108	0.895	0.105	0.893	0.110
High school incomplete			0.730 *	0.095	0.722 *	0.094	0.646 **	0.090	0.737 *	0.097	0.658 **	0.092
High school complete			0.486 **	0.064	0.482 **	0.064	0.461 **	0.064	0.489 **	0.065	0.468 **	0.065
Tertiary			0.962	0.232	0.988	0.240	0.903	0.228	0.973	0.236	0.892	0.224
**Employment Status**												
Unemployed			*Ref*		*Ref*		*Ref*		*Ref*		*Ref*	
Employed			0.871	0.091	0.855	0.090	0.916	0.103	0.838	0.089	0.904	0.102
**2011 Area Deprivation Quintile**												
Q2					*Ref*		*Ref*					
Q3					1.422	0.509	1.541	0.606				
Q4					0.833	0.291	0.984	0.376				
**Body Mass Index**												
Normal weight 18.5–24.9 kg/m2							*Ref*				*Ref*	
Underweight < 18.5 kg/m2							0.631 **	0.096			0.636 **	0.096
Overweight 25–29.9 kg/m2							1.293 *	0.154			1.300 *	0.155
Obese > = 30 kg/m2							1.616 **	0.191			1.631 **	0.192
**Diabetes**												
No current or previous diabetes diagnosis							*Ref*				*Ref*	
Current or previous diabetes diagnosis							7.119 **	1.054			7.142 **	1.056
**2001 to 2011 Area Deprivation Quintile Change**												
No change in quintile									*Ref*		*Ref*	
Quintile worsened									1.593 **	0.223	1.369 *	0.206

^*^ *p* < 0.05

^**^ *p* < 0.01

In model 2, compared to adults with no formal education, those with some high school education (OR 0.73, *p* = 0.016) and those who completed high school (OR 0.486, *p* < 0.001) have significantly lower odds of hypertension awareness. Employment status is not significantly associated with hypertension awareness. In model 3a, area deprivation is unrelated to hypertension awareness and the earlier noted protective associations between education status and hypertension awareness persist in this model. With the inclusion of BMI and diabetes in model 4a, disparities in the odds of hypertension awareness between Black South Africans with no formal education and those with some high school education (OR 0.646, *p* = 0.002) and those who completed high school (OR 0.461, *p* < 0.001) increase by 34% and 6%, respectively. There is a positive association between BMI and hypertension awareness, but a diagnosis of diabetes is unrelated to hypertension awareness.

In model 3b, residing in areas of increasing deprivation is associated with nearly 60% higher odds of hypertension awareness, compared to those living in wards whose quintiles did not change (OR 1.593, *p* = 0.001). This association is reduced (by 33%) but remains significant when adjusted for BMI and diabetes in model 4a.

#### Hypertension treatment

Table 4 displays the results of the logistic regression models for the odds of receiving treatment for hypertension, conditional upon being aware (previously diagnosed) (*n* = 2,324). Across these models, we find that increased age was significantly associated with treatment for hypertension and that females had significantly higher odds of receiving treatment than men. We did not observe any significant individual-level SES-related disparities in hypertension treatment in any of the models. While area deprivation was unrelated to hypertension treatment in model 3a, residing in areas with increasing area deprivation (model 3b) was associated with lower odds of receiving treatment for one’s hypertension (OR 0.54, *p* = 0.001), compared to living in areas with no change in area deprivation. With the addition of BMI and diabetes in Model 4b, this disparity remained significant but was reduced by 6% (OR 0.558, *p* < 0.001).

**Table 4:** Odds Ratios (OR) and Standard Errors (SE) for Hierarchical Logistic Regression Models for Hypertension Treatment (*n* = 2,324)

**Hypertension Treatment**	Model 1: Age + Sex		Model 2: Model 1 + SES		Model 3a: Model 2 + Area Deprivation Quintile		Model 4a: Model 3a + BMI and DM Prevalence		Model 3b: Model 2 + Area Deprivation Change		Model 4b: Model 3b + BMI and DM Prevalence	
**Variables**	OR	SE	OR	SE	OR	SE	OR	SE	OR	SE	OR	SE
**Age**												
31–60	*Ref*		*Ref*		*Ref*		*Ref*		*Ref*		*Ref*	
18–30	0.090 **	0.019	0.098 **	0.021	0.097 **	0.021	0.103 **	0.022	0.097 **	0.021	0.103 **	0.022
Over 60	4.348 **	0.652	4.038 **	0.635	4.026 **	0.634	4.257 **	0.677	3.942 **	0.623	4.144 **	0.661
**Sex**												
Male	*Ref*		*Ref*		*Ref*		*Ref*		*Ref*		*Ref*	
Female	1.579 **	0.228	1.569 **	0.227	1.594 **	0.232	1.391 *	0.209	1.623 **	0.237	1.415 *	0.213
**Education**												
None			*Ref*		*Ref*		*Ref*		*Ref*		*Ref*	
Some primary education			1.132	0.183	1.139	0.185	1.227	0.201	1.103	0.180	1.188	0.196
High school incomplete			0.873	0.161	0.874	0.162	0.931	0.174	0.856	0.159	0.906	0.171
High school complete			0.725	0.143	0.728	0.144	0.748	0.150	0.717	0.142	0.736	0.147
Tertiary			1.013	0.347	1.009	0.346	1.056	0.367	1.029	0.354	1.074	0.374
**Employment Status**												
Unemployed			*Ref*		*Ref*		*Ref*		*Ref*		*Ref*	
Employed			0.919	0.140	0.928	0.142	0.879	0.137	0.973	0.151	0.922	0.145
**2011 Area Deprivation Quintile**												
Q2					*Ref*		*Ref*					
Q3					0.742	0.368	0.752	0.382				
Q4					0.924	0.443	0.909	0.446				
**Body Mass Index**												
Normal weight 18.5–24.9 kg/m2							*Ref*				*Ref*	
Underweight < 18.5 kg/m2							0.725	0.163			0.743	0.169
Overweight 25–29.9 kg/m2							1.183	0.200			1.185	0.201
Obese > = 30 kg/m2							1.715 **	0.278			1.721 **	0.279
**Diabetes**												
No current or previous diabetes diagnosis							*Ref*				*Ref*	
Current or previous diabetes diagnosis							0.835	0.109			0.871	0.115
**2001 to 2011 Area Deprivation Quintile Change**												
No change in quintile									*Ref*		*Ref*	
Quintile worsened									0.540 **	0.088	0.558 **	0.093

^*^ *p* < 0.05

^**^ *p* < 0.01

#### Hypertension control

Finally, in Table 5, we analyze the log odds of controlling one’s hypertension with medication (*n* = 1,928). Across all models, we did not observe significant differences in hypertension control by age or sex.

**Table 5:** Odds Ratios (OR) and Standard Errors (SE) for Hierarchical Logistic Regression Models for Hypertension Control (*n* = 1,928)

**Hypertension Control**	Model 1: Age + Sex		Model 2: Model 1 + SES		Model 3a: Model 2 + Area Deprivation Quintile		Model 4a: Model 3a + BMI and DM Prevalence		Model 3b: Model 2 + Area Deprivation Change		Model 4b: Model 3b + BMI and DM Prevalence	
**Variables**	OR	SE	OR	SE	OR	SE	OR	SE	OR	SE	OR	SE
**Age**												
31–60	*Ref*		*Ref*		*Ref*		*Ref*		*Ref*		*Ref*	
18–30	1.837	0.677	1.728	0.643	1.723	0.642	1.652	0.617	1.747	0.651	1.680	0.627
Over 60	0.936	0.087	0.909	0.091	0.915	0.092	0.913	0.093	0.90	0.091	0.899	0.091
**Sex**												
Male	*Ref*		*Ref*		*Ref*		*Ref*		*Ref*		*Ref*	
Female	1.201	0.143	1.191	0.143	1.190	0.143	1.235	0.152	1.207	0.145	1.250	0.154
**Education**												
None			*Ref*		*Ref*		*Ref*		*Ref*		*Ref*	
Some primary education			0.970	0.105	0.969	0.105	0.967	0.106	0.959	0.104	0.957	0.105
High school incomplete			1.377 *	0.219	1.371 *	0.218	1.390 *	0.223	1.365	0.217	1.382 *	0.222
High school complete			1.195	0.223	1.198	0.225	1.194	0.225	1.193	0.223	1.189	0.223
Tertiary			0.950	0.282	0.961	0.285	0.960	0.286	0.953	0.282	0.948	0.282
**Employment Status**												
Unemployed			*Ref*		*Ref*		*Ref*		*Ref*		*Ref*	
Employed			0.716 *	0.101	0.723 *	0.102	0.714 *	0.102	0.726 *	0.103	0.717 *	0.103
**2011 Area Deprivation Quintile**												
Q2					*Ref*		*Ref*					
Q3					0.438	0.190	0.403 *	0.176				
Q4					0.441	0.189	0.399 *	0.172				
**Body Mass Index**												
Normal weight 18.5–24.9 kg/m2							*Ref*				*Ref*	
Underweight < 18.5 kg/m2							0.876	0.168			0.880	0.169
Overweight 25–29.9 kg/m2							0.898	0.120			0.906	0.120
Obese > = 30 kg/m2							0.842	0.101			0.851	0.103
**Diabetes**												
No current or previous diabetes diagnosis							*Ref*				*Ref*	
Current or previous diabetes diagnosis							0.764 **	0.075			0.784 *	0.076
**2001 to 2011 Area Deprivation Quintile Change**												
No change in quintile									*Ref*		*Ref*	
Quintile worsened									0.792	0.116	0.807	0.118

^*^ *p* < 0.05

^**^ *p* < 0.01

In model 2, higher odds of having one’s hypertension controlled are evident among those with some high school education completed, compared to those with no formal education (OR 1.377, *p* = 0.044). In contrast, Black South Africans who were employed have significantly lower odds of having their hypertension controlled than those who were unemployed (OR 0.716, *p* = 0.018). In model 3a, area deprivation is not significantly associated with hypertension control, but the patterns noted for individual-level SES remained significant when area deprivation is added to the model.

In model 4a, with the addition of BMI and diabetes, the direction, strength, and significance of the associations of high school completion and employment with hypertension control persisted, with little change to the parameter estimates. In this model, compared to Black South Africans living in the least deprived wards (2^nd^ quintile), those living in more deprived areas have significantly lower odds of having their hypertension controlled (Q3 vs Q2: OR 0.403, *p* = 0.038; Q4 vs Q2: OR 0.399, *p* = 0.033).

In model 3b, the hypertension control gap between Black South Africans with no formal education and some high school education is no longer significant. The differences in hypertension control by employment status remained significant and the magnitude of this association did not change appreciably. Change in area deprivation was not significantly associated with hypertension control.

In model 4b, we observe significant protective associations between some high school education and hypertension control (OR 1.382, *p* = 0.044), and the differences in hypertension control by employment status remained significant. Control of one’s hypertension was not significantly associated with a change in area deprivation.

## Discussion

This is one of the few studies to investigate individual and area-based socioeconomic factors linked to not only hypertension prevalence, but also levels of hypertension awareness, treatment, and control among Black South Africans. It showed that level of education was inversely related to the odds of hypertension prevalence – a pattern that has been repeatedly documented around the world and is most pronounced in LMICs like South Africa [44, 45]. Furthermore, socioeconomic disparities in secondary prevention of hypertension were also the widest in these countries, suggesting that this relationship could be driven by differences in access to healthcare [44]. Other research in South Africa has posited that individuals of higher SES are also protected from acute negative economic events and the stress that accompanies them; with consequently lower prevalence of hypertension [46].

Surprisingly, this study demonstrated that those with no education were more likely to be aware of their hypertension than those with some level of education. This association was also observed in a nationally representative sample of South Africans, wherein the authors argued that individuals with low education are likely recipients of targeted public health interventions and therefore have more frequent interactions with primary healthcare services [13]. In addition, those with no education are more likely to be at home and accessible to community healthcare workers who conduct home visits, measure their blood pressure, and educate them about hypertension.

Education was unrelated to the receipt of treatment for one’s hypertension. This finding is unanticipated, yet consistent with existing research in South Africa conducted among rural Black South Africans [33]. In this earlier study, as well as ours, the samples were drawn from populations of primarily lower SES adults, residing in more deprived areas, with limited variation in educational status in the sample. It may be that in communities like this, individuals with low education were likely to receive treatment through outreach from community health workers, while those of higher education were more likely to receive it through general practitioners using health insurance or out-of-pocket expenditures. Future research should assess the extent to which this pattern of hypertension treatment by education persists in samples of Black South Africans that are more heterogeneous concerning educational attainment.

A final intriguing education finding was that individuals with some secondary education had higher levels of control of hypertension than those with no education; but adults who completed primary, secondary, and tertiary education did not differ from those with no education in the control of hypertension. A study conducted among a sample of low-SES Black South Africans attending primary care facilities in the Western Cape had similar results in that only secondary education was predictive of hypertension control [47]. Prior nationally-representative research in South Africa has also found similar associations between secondary education and hypertension control among women [15, 18]. While these findings were largely attributed to the increases in health literacy that accompany a secondary school education, it may be that educational awareness of hypertension does not necessarily translate into an understanding of the value of early treatment, the consequences of treatment delay, and the benefits from treatment in general.

When considering why Black South Africans in our study who had a tertiary level of education did not differ in their odds of controlling their hypertension compared to those with no education, several avenues for future research can be considered. In the context of the community under study, people with higher levels of education often commute to urban areas to work long hours, in poorly paid jobs that have high levels of job-related stressors, including racial discrimination, and limited prospects for advancement [37]. The negative health effects of these job-related stressors and the unhealthy copying strategies that accompany them have received some attention in the South African context, however, more sustained research efforts have been conducted in the United States [48–51]. Furthermore, in our sample, we found that employed Black South Africans had lower odds of hypertension control compared to their unemployed counterparts. It may be that work-related demands and stressors may challenge the initiation and maintenance of the treatment of hypertension for employed adults, especially those who lack the time to access health services regularly due to their work commitments. Indeed, another study of Black South Africans in KwaZulu-Natal found that employed individuals had lower odds of being screened for hypertension by community health workers, with the authors suggesting that these individuals were less likely to be present at the time of data collection due to their work commitments [52]. This association has been observed nationally as well, the WHO-SAGE study found that employed individuals were less likely to be surveyed during at-home data collection procedures [13].

However plausible these potential explanations for our results may be, future work should investigate whether exposure to work-related stressors and demands is the etiological agent for our observed associations between education, employment status, and hypertension outcomes - and whether this association holds in different South African contexts. Outside of South Africa, the non-equivalence of socioeconomic status indicators across races, and their health benefits is well documented in scientific literature [53–55].

While other studies have investigated the association of poverty with hypertension in South Africa; this study is unique in its investigation of the association between hypertension risk and a multidimensional measure of social and economic deprivation at the ward level, both cross-sectionally and over time. Our results indicated that individuals living in more deprived areas had significantly higher odds of hypertension prevalence and lower odds of hypertension control. To our knowledge, only one other study has assessed the relationship between this measure of area-level deprivation with cardiovascular health outcomes. In that analysis, which used data from SANHANES-1, area-level deprivation was positively associated with the prevalence of ischemic heart disease, but not hypertension [43].

Environmental exposures, such as air pollution, industrial toxins, and contaminated water may contribute to some of the observed associations between area-level deprivation and hypertension. Existing research from other LMICs has proposed that exposure to air pollution mediates some of the association between neighborhood deprivation and hypertension [56]. In South Africa, the stark racial patterning of urban and rural geographies that resulted from Apartheid-era policies such as the Group Areas Act, combined with the unregulated industrial mining complexes that surrounded them, have ensured that areas that have been disproportionately impacted by environmental pollutants *were and continue to be* those primarily occupied by Black South Africans [57–59]. One such contemporary example in the literature on environmental racism in South Africa is the impact of the Durban South Industrial Basin on the disproportionately high rates of respiratory illness experienced by the primarily Black South African population nearby [60]. Although we were unable to assess whether our observed associations were caused by exposure to environmental stressors, future empirical inquiries in South Africa should aim to examine the relationship between hypertension outcomes and environmental stressors unique to Black South African neighborhoods.

Other potential explanations for these observed associations between area-level deprivation and hypertension risk are related to the changes in nutrition and lifestyle that accompany the epidemiological transition. Conceptualized by Abdel Omran, the epidemiological transition is a theory that seeks to explain the shift in the prevalence and burden of disease from primarily infectious diseases to NCDs that accompanies urbanization, technological modernization, and advancements in medicine [19]. As a result of this transition, South African diets increased reliance on highly processed foods due to accelerated marketing and their increased availability over a relatively short time, supporting the creation of ‘obesogenic’ environments, which are strongly associated with hypertension [61, 62] [63, 64]. The negative effects of these changes in nutrition are compounded by declining physical activity due to changing lifestyles and work patterns. However, individuals in more deprived areas may be further at risk due to the non-availability of resources and facilities for sports and exercise [65]. Physical activity for these individuals is usually confined to walking and household chores which can include tending to home gardens or livestock. Furthermore, deprived areas often lack green spaces for communal activity and recreation, which have well-documented health benefits and have been shown to mitigate the negative health effects of environmental pollutants in deprived communities [66–70].

Concerning the change in area-level deprivation, our results indicated that hypertensive individuals residing in places that had become more deprived from 2001 to 2011 had higher odds of being aware of their condition yet lower odds of receiving treatment for it. The higher awareness might reflect that in areas of increasing deprivation, there was greater outreach from the community health workers over time which could have led to increased diagnoses in these communities. Future research in South Africa is needed to elucidate why individuals who are the recipients of such campaigns may not experience the desired outcomes concerning treatment and control. A mixed-methods study of rural South Africans found that although study participants had intermediate to high knowledge about hypertension, qualitative data highlighted structural barriers preventing treatment, such as distance to healthcare institutions, inability to afford more nutritious foods, and exposure to social stressors, including interpersonal conflict due to economic stress [71]. Although South Africa has been well-intentioned and ambitious in its attempts to increase access to primary care in rural settings, several barriers exist to successful implementation. One such barrier is the pervasive staffing shortages in these healthcare facilities, which not only results in gaps in care; but makes some individuals reluctant to utilize these facilities, especially individuals who have the means to access well-staffed hospitals [72].

## Conclusions

Our findings can inform policies and interventions aiming to improve the health of Black South Africans living in these communities, such as screening programs, health education, and health promotion interventions. Our study found that Black South Africans in our sample who had low levels of education and lived in deprived areas were at disproportionately high risk for hypertension. In addition to health education programs designed to reduce the nutritional and lifestyle risk factors for hypertension in these communities, interventions aimed to reduce the structural barriers to hypertension prevention should be considered. One such example is the Centralised Chronic Medication Dispensing and Distribution Programme by the KwaZulu-Natal Department of Health, which delivers medication to households, workplaces, or community-based depots to expand access to healthcare to those who have historically and currently face barriers to care [73]. According to a systematic review of NCD prevention programs in LMICs, empowerment-based educational programs in South Africa have reduced patients’ HbA1c across several years of follow-up, which may inform similar initiatives designed to reduce blood pressure [74, 75]. Additionally, research conducted across urban and rural geographies in Cameroon has demonstrated that shifting care to non-physician clinician (NPC) facilities resulted in significantly reduced blood pressure among the patients at these clinics [76, 77]. For Black South Africans already diagnosed with hypertension, tailored and targeted health counseling following a hypertension diagnosis may enhance treatment and control, as has been found in previous NCD screening programs in the United States [78, 79]. Counseling programs in South Africa can also be informed by existing human immunodeficiency virus (HIV) interventions, where HIV counseling services and linkage strategies led to improved treatment after HIV diagnoses [80].

We acknowledge the multiple limitations of our analyses presented in this paper. Despite its size and wide geographic catchment area, our sample was purposively selected without the utilization of probability sampling, and as such, there is potential for selection bias to impact our observed results. We recognize that our obtained sample was drawn from individuals living in wards that were on average, more deprived. While this reduces the generalizability of our findings to outside our analytic sample, it should be noted that Black South Africans are more likely than other racial groups to reside in more deprived areas and that these findings shed light on challenges faced by a large proportion of Black South Africans [81]. The analysis presented in this manuscript is also limited in that it does not include household-level data, because it was not collected as part of the HealthRise study. Without household-level indicators, more robust analyses which could better distinguish socioeconomic factors at the individual and area level, including multi-level modeling, could not be conducted. Finally, our data are cross-sectional, and therefore none of our reported associations should be interpreted as causally related. Despite its limitations, this study is one of the first to examine area-level determinants of hypertension among Black South Africans and the results of this analysis can inform future epidemiological research to better understand their individual-level and area-level correlates of hypertension risk. This study can also inform targeted public health interventions and policy initiatives aimed at improving hypertension detection, awareness, treatment, and control in South Africa.

## Supplementary Information

Below is the link to the electronic supplementary material.

**Additional file 1: Table S1.** Ward & Municipality. **Table S2.** Location & 2001 Area Deprivation Quintile. **Table S3.** Location & 2011 Area Deprivation Quintile. **Table S4.** 2001 Area Deprivation Quintile & 2011 Area Deprivation Quintile. **Table S5.** Location and Area Deprivation Quintile Change.

## Data Availability

The datasets generated and/or analyzed during the current study are not publicly available but are available from the corresponding author on reasonable request.

## Abbreviations

NCDs: Noncommunicable diseases
LMIC: Low- and middle-income countries
CVD: Cardiovascular disease
DHS: Demographic and Health Survey
SANHANES: South African National Health and Nutrition Examination Survey
WHO-SAGE: World Health Organization’s Study on Global Ageing
SES: Socioeconomic status
NIDS: National Income Dynamics Survey
NGO: Non-governmental organization
SETA: Health & Welfare Sector Education Training Authority
IHME: Institute for Health Metrics and Evaluation
CHWs: Community health workers
APC: Adult Primary Care
SAMPI: South African Multidimensional Poverty Index
BMI: Body mass index
HIV: Human immunodeficiency virus

## References

[1] Noncommunicable Diseases Fact Sheet. 2021, World Health Organization.

[2] Kabudula CW (2017). Progression of the epidemiological transition in a rural South African setting: findings from population surveillance in Agincourt, 1993–2013. BMC Public Health.

[3] Kabudula CW (2021). Mortality transition over a quarter century in rural South Africa: findings from population surveillance in Agincourt 1993–2018. Glob Health Action.

[4] Noncommunicable Diseases (NCD) Country Profiles - South Africa. 2014, World Health Organization.

[5] Bromfield S, Muntner P (2013). High Blood Pressure: The Leading Global Burden of Disease Risk Factor and the Need for Worldwide Prevention Programs. Curr Hypertens Rep.

[6] Fact Sheet: Cardiovascular diseases in South Africa. 2017, World Heart Federation.

[7] Shisana O (2014). The South African National Health and Nutrition Examination Survey, 2012: SANHANES-1: the health and nutritional status of the nation.

[8] Zhou B (2021). Worldwide trends in hypertension prevalence and progress in treatment and control from 1990 to 2019: a pooled analysis of 1201 population-representative studies with 104 million participants. Lancet.

[9] Gupta RD, et al., Prevalence and associated factors of hypertension among South African adults: findings from the Demographic and Health Survey 2016. J Public Health, 2021.

[10] Steyn K (2001). Hypertension in South African adults: results from the Demographic and Health Survey, 1998. J Hypertens.

[11] Siko PR, van Deventer C (2017). Compliance with standard treatment guidelines in the management of hypertension: a review of practice of healthcare workers in Potchefstroom, North West Province, South Africa. South Afr Fam Pract.

[12] Berry KM (2017). Quantifying unmet need for hypertension care in South Africa through a care cascade: evidence from the SANHANES, 2011–2012. BMJ Glob Health.

[13] Ware LJ (2019). Predictors of hypertension awareness, treatment and control in South Africa: results from the WHO-SAGE population survey (Wave 2). J Hum Hypertens.

[14] Kandala NB (2021). Mapping the Burden of Hypertension in South Africa: A Comparative Analysis of the National 2012 SANHANES and the 2016 Demographic and Health Survey. Int J Environ Res Public Health.

[15] Thomas R, Burger R, Hauck K (2018). Richer, wiser and in better health? The socioeconomic gradient in hypertension prevalence, unawareness and control in South Africa. Soc Sci Med.

[16] Pillay-van Wyk V (2016). Mortality trends and differentials in South Africa from 1997 to 2012: second National Burden of Disease Study. Lancet Glob Health.

[17] Weimann A, Dai D, Oni T (2016). A cross-sectional and spatial analysis of the prevalence of multimorbidity and its association with socioeconomic disadvantage in South Africa: A comparison between 2008 and 2012. Soc Sci Med.

[18] Cois A, Ehrlich R (2014). Analysing the socioeconomic determinants of hypertension in South Africa: a structural equation modelling approach. BMC Public Health.

[19] Omran AR (2005). The epidemiologic transition: a theory of the epidemiology of population change. 1971. Milbank Q.

[20] McKeown RE (2009). The Epidemiologic Transition: Changing Patterns of Mortality and Population Dynamics. Am J Lifestyle Med.

[21] Leibbrandt M (2010). Trends in South African Income Distribution and Poverty since the Fall of Apartheid.

[22] Tregenna F, Tsela M (2012). Inequality in South Africa: The distribution of income, expenditure and earnings. Dev South Afr.

[23] Leibbrandt M, Finn A, Woolard I (2012). Describing and decomposing post-apartheid income inequality in South Africa. Dev South Afr.

[24] Harris B (2011). Inequities in access to health care in South Africa. J Public Health Policy.

[25] McLaren Z, Ardington C, Leibbrandt M (2013). Distance as a barrier to health care access in South Africa.

[26] Reddy SP (2021). Race, geographical location and other risk factors for hypertension: South African National Health and Nutrition Examination Survey 2011/12. SSM Popul Health.

[27] van Rooyen JM (2000). An epidemiological study of hypertension and its determinants in a population in transition: the THUSA study. J Hum Hypertens.

[28] Peer N (2013). A High Burden of Hypertension in the Urban Black Population of Cape Town: The Cardiovascular Risk in Black South Africans (CRIBSA) Study. PLoS ONE.

[29] Steyn K (1996). Hypertension in the black community of the Cape Peninsula, South Africa. East Afr Med J.

[30] Kandala N-B (2013). Geographic Variation of Hypertension in Sub-Saharan Africa: A Case Study of South Africa. Am J Hypertens.

[31] Zatu MC (2016). Alcohol intake, hypertension development and mortality in black South Africans. Eur J Prev Cardiol.

[32] Mphekgwana PM (2020). Hypertension Prevalence and Determinants among Black South African Adults in Semi-Urban and Rural Areas. Int J Environ Res Public Health.

[33] Sharma JR (2021). Prevalence of Hypertension and Its Associated Risk Factors in a Rural Black Population of Mthatha Town, South Africa. Int J Environ Res Public Health.

[34] HealthRise South Africa: Improving care for people living with cardiovascular disease, diabetes. 2016, HealthRise.

[35] Wollum A (2018). Identifying gaps in the continuum of care for cardiovascular disease and diabetes in two communities in South Africa: Baseline findings from the HealthRise project. PLoS ONE.

[36] Massyn N, et al. District Health Barometer 2016/17. 2017, Health Systems Trust.

[37] Madela S (2020). Early detection, care and control of hypertension and diabetes in South Africa: A community-based approach. Afr J Prim Health Care Fam Med.

[38] Jardim TV (2017). Hypertension management in a population of older adults in rural South Africa. J Hypertens.

[39] Strategic plan for the prevention and control of non-communicable disease 2013–2017. Pretoria: National Department of Health. 2012

[40] Primary care 101 guideline 2013/14: Symptom-based integrated approach to the adult in primary care. Pretoria: National Department of Health. 2013.

[41] The South African MPI: Creating a multidimensional poverty index using Census data. Pretoria: Statistics South Africa. 2014.

[42] Oguttu JW, Ncayiyana JR (2020). Social capital and self-rated health of residents of Gauteng province: Does area-level deprivation influence the relationship?. SSM Popul Health.

[43] Dwane N, Wabiri N, Manda S (2020). Small-area variation of cardiovascular diseases and select risk factors and their association to household and area poverty in South Africa: Capturing emerging trends in South Africa to better target local level interventions. PLoS ONE.

[44] Rosengren A (2019). Socioeconomic status and risk of cardiovascular disease in 20 low-income, middle-income, and high-income countries: the Prospective Urban Rural Epidemiologic (PURE) study. Lancet Glob Health.

[45] Kirschbaum TK (2022). The Association of Socioeconomic Status With Hypertension in 76 Low- and Middle-Income Countries. J Am Coll Cardiol.

[46] Gangaidzo T, et al. Stressful life events, neighbourhood characteristics, and systolic blood pressure in South Africa. J Hum Hypertens, 2022. 10.1038/s41371-022-00695-9 35513441

[47] Folb N (2016). Socioeconomic and modifiable predictors of blood pressure control for hypertension in primary care attenders in the Western Cape, South Africa. S Afr Med J.

[48] Ren XS, Amick BC, Williams DR (1999). Racial/ethnic disparities in health: the interplay between discrimination and socioeconomic status. Ethn Dis.

[49] Bernard DL, Jones SCT, Volpe VV (2020). Impostor Phenomenon and Psychological Well-Being: The Moderating Roles of John Henryism and School Racial Composition Among Black College Students. J Black Psychol.

[50] Peer N (2020). A high burden of adverse life events and poor coping mechanisms experienced by urban-dwelling black South Africans. PLoS ONE.

[51] Peltzer K (2009). Job stress, job satisfaction and stress-related illnesses among South African educators. Stress Health J Int Soc Invest Stress.

[52] Chikafu H, Chimbari M (2021). Hypertension care cascade in the Ingwavuma rural community, uMkhanyakude District, KwaZulu-Natal province of South Africa. PeerJ.

[53] Williams DR (1996). Race/Ethnicity and Socioeconomic Status: Measurement and Methodological Issues. Int J Health Serv.

[54] Williams DR (1999). Race, Socioeconomic Status, and Health The Added Effects of Racism and Discrimination. Ann N Y Acad Sci.

[55] Bell CN (2020). Racial Non-equivalence of Socioeconomic Status and Self-rated Health among African Americans and Whites. SSM Popul Health.

[56] Schutte AE (2021). Hypertension in Low- and Middle-Income Countries. Circ Res.

[57] Ruiters G (2001). Environmental racism and justice in South Africa’s transition. Politikon.

[58] Leonard L (2018). Mining Corporations, Democratic Meddling, and Environmental Justice in South Africa. Soc Sci.

[59] Kemp-Neal WCC. Environmental Racism: Using Environmental Planning to Lift People Out of Poverty, and Re-shape the Effects of Climate Change & Pollution in Communities of Color. Fordham Envtl L Rev 2021. 32(3).

[60] Dlamini ET, Mngoma LW, Dlamini ET (2014). Environmental Racism In The Durban South Region : The Importance of Public Participation in Environmental Impact Assessments. OIDA Int J Sustain Dev..

[61] McHiza ZJ (2013). Content analysis of television food advertisements aimed at adults and children in South Africa. Public Health Nutr.

[62] Ryu S (2019). Secular trends in the association between obesity and hypertension among adults in the United States, 1999–2014. Eur J Intern Med.

[63] Popkin BM, Adair LS, Ng SW (2012). Global nutrition transition and the pandemic of obesity in developing countries. Nutr Rev.

[64] Modjadji P, Madiba S (2019). The double burden of malnutrition in a rural health and demographic surveillance system site in South Africa: a study of primary schoolchildren and their mothers. BMC Public Health.

[65] McVeigh JA, Norris SA, de Wet T (2004). The relationship between socio-economic status and physical activity patterns in South African children. Acta Paediatr.

[66] Rigolon A (2021). Green Space and Health Equity: A Systematic Review on the Potential of Green Space to Reduce Health Disparities. Int J Environ Res Public Health.

[67] Matthew McConnachie M, Shackleton CM (2010). Public green space inequality in small towns in South Africa. Habitat Int.

[68] Donaldson R (2016). Access to the urban national park in Cape Town: Where urban and natural environment meet. Habitat Int.

[69] Willemse L (2013). A Flowmap–geographic information systems approach to determine community neighbourhood park proximity in Cape Town. S Afr Geogr J.

[70] Shackleton CM, Blair A (2013). Perceptions and use of public green space is influenced by its relative abundance in two small towns in South Africa. Landsc Urban Plan.

[71] Jongen VW (2019). Hypertension in a rural community in South Africa: what they know, what they think they know and what they recommend. BMC Public Health.

[72] Maphumulo WT, Bhengu BR (2019). Challenges of quality improvement in the healthcare of South Africa post-apartheid: A critical review. Curationis.

[73] Bogart LM (2022). Implementation of South Africa’s Central Chronic Medicine Dispensing and Distribution Program for HIV Treatment: A Qualitative Evaluation. AIDS Behav.

[74] Kane J (2017). A systematic review of primary care models for non-communicable disease interventions in Sub-Saharan Africa. BMC Fam Pract.

[75] Price C (2011). Long-term glycaemic outcome of structured nurse-led diabetes care in rural Africa. QJM.

[76] Labhardt ND (2010). Task shifting to non-physician clinicians for integrated management of hypertension and diabetes in rural Cameroon: a programme assessment at two years. BMC Health Serv Res.

[77] Kengne AP (2009). Setting-up nurse-led pilot clinics for the management of non-communicable diseases at primary health care level in resource-limited settings of Africa. Pan Afr Med J.

[78] Fairchild C (1979). Hypertension Counseling. Occup Health Nurs.

[79] Reisin E (1978). Effect of weight loss without salt restriction on the reduction of blood pressure in overweight hypertensive patients. N Engl J Med.

[80] Sharma M (2015). Systematic review and meta-analysis of community and facility-based HIV testing to address linkage to care gaps in sub-Saharan Africa. Nature.

[81] Gradín C (2012). Race, Poverty and Deprivation in South Africa. J Afr Econ.

