# Supplementary material for: Individual and area-level socioeconomic correlates of hypertension prevalence, awareness, treatment, and control in uMgungundlovu, KwaZulu-Natal, South Africa
Source: BMC Public Health. 2023 Mar 2;23:417. doi: 10.1186/s12889-023-15247-0 (PMC9979474; doi:10.1186/s12889-023-15247-0)
Supplement: Supplementary file 1 — Additional file 1: Table S1. Ward & Municipality. Table S2. Location & 2001 Area Deprivation Quintile. Table S3. Location & 2011 Area Deprivation Quintile. Table S4. 2001 Area Deprivation Quintile & 2011 Area Deprivation Quintile. Table S5. Location and Area Deprivation Quintile Change. [file 12889_2023_15247_MOESM1_ESM.docx]

**Table S1 – Ward & Municipality**

|  | **Municipality** | | | | |
| --- | --- | --- | --- | --- | --- |
| **Ward** | Msunduzi | uMkhambathini | uMshwathi | Missing | Total |
| Ward 1 | 64 | 837 | 784 | 0 | 1,685 |
| Ward 8 | 0 | 0 | 1,294 | 0 | 1,294 |
| Ward 11 | 2,428 | 0 | 0 | 0 | 2,428 |
| Ward 31 | 816 | 0 | 0 | 0 | 816 |
| Ward 34 | 182 | 0 | 0 | 0 | 182 |
| Missing | 895 | 0 | 3 | 0 | 898 |
| Total | 4,385 | 837 | 2,081 | 0 | 7,303 |

**Table S2 – Location & 2001 Area Deprivation Quintile**

|  | **2001 Area Deprivation Quintile** | | | | | | |
| --- | --- | --- | --- | --- | --- | --- | --- |
| **Municipality and Ward** | Q1-Least Deprived | Q2 | Q3 | Q4 | Q5-Most Deprived | Missing | Total |
| Msunduzi Ward 1 | 0 | 64 | 0 | 0 | 0 | 0 | 64 |
| Msunduzi Ward 11 | 0 | 0 | 0 | 2,428 | 0 | 0 | 2,428 |
| Msunduzi Ward 31 | 816 | 0 | 0 | 0 | 0 | 0 | 816 |
| Msunduzi Ward 34 | 0 | 182 | 0 | 0 | 0 | 0 | 182 |
| uMkhambathini Ward 1 | 0 | 0 | 0 | 837 | 0 | 0 | 837 |
| uMshwathi Ward 1 | 0 | 0 | 784 | 0 | 0 | 0 | 784 |
| uMshwathi Ward 8 | 0 | 0 | 0 | 1,294 | 0 | 0 | 1,294 |
| Missing | 0 | 0 | 0 | 0 | 0 | 898 | 898 |
| Total | 816 | 246 | 784 | 4,559 | 0 | 898 | 7,303 |

**Table S3 – Location & 2011 Area Deprivation Quintile**

|  | **2011 Area Deprivation Quintile** | | | | | | |
| --- | --- | --- | --- | --- | --- | --- | --- |
| **Municipality and Ward** | Q1-Least Deprived | Q2 | Q3 | Q4 | Q5-Most Deprived | Missing | Total |
| Msunduzi Ward 1 | 0 | 0 | 64 | 0 | 0 | 0 | 64 |
| Msunduzi Ward 11 | 0 | 0 | 0 | 2,428 | 0 | 0 | 2,428 |
| Msunduzi Ward 31 | 0 | 0 | 816 | 0 | 0 | 0 | 816 |
| Msunduzi Ward 34 | 0 | 182 | 0 | 0 | 0 | 0 | 182 |
| uMkhambathini Ward 1 | 0 | 0 | 0 | 837 | 0 | 0 | 837 |
| uMshwathi Ward 1 | 0 | 0 | 784 | 0 | 0 | 0 | 784 |
| uMshwathi Ward 8 | 0 | 0 | 0 | 1,294 | 0 | 0 | 1,294 |
| Missing | 0 | 0 | 0 | 0 | 0 | 898 | 898 |
| Total | 0 | 182 | 1,664 | 4,559 | 0 | 898 | 7,303 |

**Table S4 – 2001 Area Deprivation Quintile & 2011 Area Deprivation Quintile**

|  | **2011 Area Deprivation Quintile** | | | | | | |
| --- | --- | --- | --- | --- | --- | --- | --- |
| **2001 Area Deprivation Quintile** | Q1-Least Deprived | Q2 | Q3 | Q4 | Q5-Most Deprived | Missing | Total |
| Q1-Least Deprived | 0 | 0 | 816 | 0 | 0 | 0 | 816 |
| Q2 | 0 | 182 | 64 | 0 | 0 | 0 | 246 |
| Q3 | 0 | 0 | 784 | 0 | 0 | 0 | 784 |
| Q4 | 0 | 0 | 0 | 4,559 | 0 | 0 | 4,559 |
| Q5-Most Deprived | 0 | 0 | 0 | 0 | 0 | 0 | 0 |
| Missing | 0 | 0 | 0 | 0 | 0 | 898 | 898 |
| Total | 0 | 182 | 1,664 | 4,559 | 0 | 898 | 7,303 |

**Table S5 – Location and Area Deprivation Quintile Change**

|  | **Area Deprivation Quintile Change from 2001 to 2011** | | | |
| --- | --- | --- | --- | --- |
| **Municipality and Ward** | No Change | Quintile Worsened | Missing | Total |
| Msunduzi Ward 1 | 0 | 64 | 0 | 64 |
| Msunduzi Ward 11 | 2,428 | 0 | 0 | 2,428 |
| Msunduzi Ward 31 | 0 | 816 | 0 | 816 |
| Msunduzi Ward 34 | 182 | 0 | 0 | 182 |
| uMkhambathini Ward 1 | 837 | 0 | 0 | 837 |
| uMshwathi Ward 1 | 784 | 0 | 0 | 784 |
| uMshwathi Ward 8 | 1,294 | 0 | 0 | 1,294 |
| Missing | 0 | 0 | 898 | 898 |
| Total | 5,525 | 880 | 898 | 7,303 |
